# Life‐Threatening Bupropion Poisoning Successfully Treated by Intravenous Lipid Emulsion

**DOI:** 10.1155/crcc/3324437

**Published:** 2026-08-18

**Authors:** Francesca Chiara Della Casa, Vincent Haufroid, Kevin Delongie, Thomas Coppens, Ester Ponzetto, Nicolas De Schryver

**Affiliations:** ^1^ Intensive Care Unit, Saint-Pierre Hospital, Ottignies-Louvain-la-Neuve, Belgium; ^2^ Department of Clinical Chemistry, Saint-Luc University Hospital, Brussels, Belgium, saintluc.be; ^3^ Louvain Centre for Toxicology and Applied Pharmacology (LTAP), Experimental and Clinical Research Institute (IREC), UCLouvain, Brussels, Belgium; ^4^ Department of Neurology, Saint-Pierre Hospital, Ottignies-Louvain-la-Neuve, Belgium

**Keywords:** bupropion, intravenous lipid emulsion, poisoning

## Abstract

Bupropion is a synthetic cathinone that acts as a norepinephrine and dopamine reuptake inhibitor and is an atypical antidepressant commonly prescribed for major depressive disorder and smoking cessation. Although generally well tolerated at therapeutic doses, bupropion overdose can lead to severe toxicity, primarily characterized by seizures and cardiac arrhythmias. Management of severe intoxications remains mainly supportive, with no specific antidote available. Intravenous lipid emulsion (ILE) therapy has emerged as a potential treatment for poisoning with highly lipophilic drugs. Initially found to reverse local anesthetic toxicity, ILE is hypothesized to work via multiple mechanisms, including a “lipid shuttle” effect and direct stabilization of highly excitable membranes in the brain and heart. However, its precise role in nonanesthetic drug intoxications remains incompletely understood. We report a case of a massive, intentional bupropion overdose (24,000 mg) in a patient with no coingestion of other toxic substances aside from alcohol. The patient developed refractory seizures and severe ventricular arrhythmias, which were successfully reversed after ILE administration. Serial blood measurements of bupropion and its metabolites were performed before and after ILE administration, supporting both lipid sequestration of the drug and direct cardioprotective effects. A preprint of this study is available online (Della Casa et al. 2026).

## 1. Introduction

Bupropion is a widely prescribed drug for major depressive disorder and smoking cessation but is also frequently involved in cases of intentional overdose. Severe intoxication can lead to life‐threatening neurological and cardiovascular complications, including refractory seizures and malignant arrhythmias, for which treatment remains largely supportive.

Intravenous lipid emulsion (ILE) therapy has emerged as a potential adjunctive therapy in severe poisonings with lipophilic drugs, although its role in non–local anesthetic toxicity is still debated and supported by limited clinical evidence.

We report a case of massive bupropion overdose complicated by refractory seizures and ventricular arrhythmias, with a rapid and sustained clinical improvement following ILE administration. Serial measurements of drug and metabolite concentrations provide further insight into the potential mechanisms underlying its effects. A preprint of this study is available online [1].

## 2. Case Presentation

A 35‐year‐old woman with a history of alcohol abuse and depression presented to the emergency department 2 h after intentionally ingesting approximately 24,000 mg of extended‐release bupropion together with alcohol. On admission, heart rate was 137 bpm, blood pressure was 150/80 mmHg, respiratory rate was 15/min, and pulse oxygen saturation was 98% while breathing ambient air. The Glasgow Coma Scale (GCS) score was 12/15 (E4V2M6). Laboratory analysis revealed an ethanol level of 2.41 g/L, whereas the other laboratory investigations were unremarkable. She rapidly deteriorated with the GCS score falling to 6/15 (E4V1M1) and experienced a first generalized tonic–clonic seizure. She was then intubated, and activated charcoal (50 g) was administered via a nasogastric tube. She was transferred to the intensive care unit (ICU) 4.5 h after ingestion.

Upon ICU admission, she presented with two additional seizures and was treated with 5 mg diazepam followed by a continuous infusion of 0.05 mg/kg/h and levetiracetam 60 mg/kg. Continuous electroencephalogram (EEG) monitoring was initiated and showed a severe burst–suppression pattern with approximately 90% of the time in suppression and highly symmetric bursts. This pattern, characterized by alternating periods of high‐amplitude electrical activity and near‐isoelectric suppression, reflects profound but potentially reversible cortical dysfunction. General anesthesia was maintained with a continuous infusion of 3 mg/kg/h of propofol.

**Figure 1:**
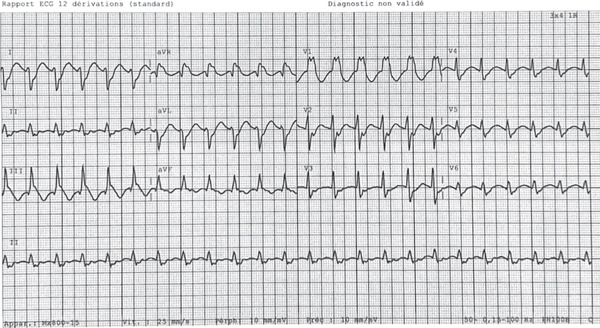
Electrocardiogram recorded after ICU admission showing sustained ventricular tachycardia.

The initial electrocardiogram (ECG) revealed sinus tachycardia at 110 bpm, with a slightly prolonged QRS duration (126 ms) and a corrected QT (QTc) interval of 470 ms. A transthoracic echocardiogram showed preserved biventricular systolic function. Blood gas analysis revealed metabolic acidosis with a pH of 7.20, pCO_2_ of 42 mmHg, bicarbonates of 16.3 mmol/L, and lactate of 5.74 mmol/L. She began to exhibit frequent polymorphic premature ventricular contractions (PVCs) that progressed to multiple episodes of incessant sustained and unsustained ventricular tachycardia (Figure 1). Blood pressure progressively decreased, and norepinephrine was started and titrated up to 0.15 mcg/kg/min. She was then given two 100‐mEq boluses of sodium bicarbonate (3.3 mEq/kg), 2 g of calcium gluconate, and 3 g of magnesium sulfate, but the arrhythmic storm persisted, and hemodynamic instability worsened. Serum sodium level rose to 151 mmol/L, precluding further significant sodium bicarbonate administration.

Considering the life‐threatening situation, ILE (Intralipid 20%) was then administered 8 h after bupropion ingestion, with a bolus of 1.5 mL/kg, followed by an infusion of 0.25 mL/kg/min during the next 3 min. Two minutes after the start of the ILE bolus, ventricular tachycardia completely resolved and did not recur.

QRS duration remained increased at 120 ms, and the QTc interval was still prolonged (460 ms), but no further PVCs were observed. Norepinephrine was discontinued during the bolus administration, and lactate levels normalized. Simultaneously, the EEG pattern showed a reduction in interburst intervals and an increase in burst amplitude, with approximately 50% of the bursts demonstrating epileptic characteristics.

Given the excellent immediate response following the bolus and the initial ILE infusion and considering the long elimination half‐life of bupropion, as well as the theoretical maximum recommended cumulative dose of ILE, the infusion was reduced after the initial bolus to 1.5 mL/kg/h (0.025 mL/kg/min) for the next 2 h. It was then gradually tapered to 45 mL/h (0.0125 mL/kg/min) during the third hour, 22 mL/h (0.00625 mL/kg/min) during the fourth hour, and 11 mL/h (0.003125 mL/kg/min) for an additional 10 h before discontinuation. The total dose administered was 500 mL (8.3 mL/kg) over 14 h.

QRS and QTc intervals gradually shortened and completely normalized within 24 h after ingestion. The patient was extubated on Day 3 and transferred to the ward. She received psychiatric support, denied any genuine suicidal intent, and was finally discharged from the hospital on Day 6.

**Figure 2:**
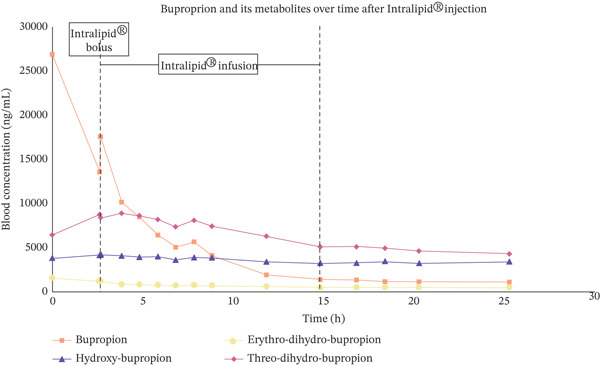
Bupropion and metabolite serum levels over time. The plasma quantification of bupropion and its three major metabolites was performed by high‐performance liquid chromatography–tandem mass spectrometry (UHPLC–MS/MS) using a Waters Aquity I Class system coupled to a Xevo TQs Triple Quadrupole instrument (Waters).

Blood samples for determination of bupropion and metabolites serum levels were obtained 2 h before the ILE bolus, immediately before the bolus, 10 min after the bolus, then hourly for the first 6 h, and at Hours 12, 15, 17, 19, 20, and 25 after the initial measurement. The evolution of the serum levels of bupropion and its metabolites over time is depicted in Figure 2.

The initial sampling 90 min before ILE administration revealed a very high serum concentration of bupropion (26,952 ng/mL) and hydroxybupropion (3788 ng/mL). A repeated sample obtained immediately before the ILE bolus showed a twofold decrease in bupropion concentration, whereas hydroxybupropion continued to increase slightly. After ILE administration, bupropion serum levels increased, whereas its metabolite levels progressively decreased over time.

Genetic testing revealed that our patient was a normal metabolizer for CYP2B6, suggesting that bupropion metabolism was not inherently impaired.

## 3. Discussion

Bupropion is a norepinephrine and dopamine reuptake inhibitor used in the treatment of depressive disorders and smoking cessation. The true incidence of bupropion poisoning is unknown, but more than 20,000 exposures are reported annually to US poison centers, making bupropion one of the most frequently implicated antidepressants in overdose [2, 3]. Bupropion toxicity is characterized by neurological and cardiovascular complications. Common symptoms include sinus tachycardia (83%), hypertension (56%), seizures (37%), gastrointestinal symptoms (37%), and agitation (32%) [4]. Bupropion cardiotoxicity is commonly associated with QRS widening, QTc prolongation, and, in severe cases, myocardial depression. As with most QT‐prolonging drugs, QTc prolongation is primarily caused by inhibition of the rapid component of the delayed rectifier potassium current (IKr). In contrast, QRS widening does not appear to result predominantly from classic fast sodium channel blockade but is thought to be mainly related to inhibition of myocardial gap junctions [5]. This hypothesis is further supported by the frequently reported limited efficacy of sodium bicarbonate in bupropion overdose, as in our patient.

The recommended daily dose of bupropion ranges from 150 to 300 mg. The drug is metabolized mainly by the cytochrome P450 2B6 (CYP2B6) into hydroxybupropion, its main active metabolite. Additional metabolism via CYP3A4 and CYP2C19 produces threo‐ and erythro‐dihydrobupropion, which retain 20%–50% of the parent drug′s pharmacological activity. Oral administration of 300 mg of extended‐release bupropion results in a maximal serum concentration (Cmax) of approximately 160 ng/mL after 5 h, whereas metabolites peak between 7 and 8 h. Therapeutic serum concentrations of hydroxybupropion are three to 14 times higher than those of bupropion (600–2000 ng/mL). Elimination occurs mainly via the kidneys, with 87% excreted in urine and about 10% in feces. The elimination half‐life is approximately 21 h for bupropion and hydroxybupropion.

Management of bupropion poisoning is mainly supportive and aims to stabilize hemodynamics and control seizures while awaiting endogenous drug elimination. Activated charcoal may reduce absorption if administered within 1–2 h after ingestion [6]. Sodium bicarbonate is recommended in case of cardiovascular toxicity, as for other forms of intoxications with sodium channel blockers, typically characterized by QRS widening on ECG [7]. In the absence of a specific antidote and when these conservative measures fail to stabilize the hemodynamics, the lipophilic profile of bupropion with its high octanol/water partition coefficient (log*P* 3.47) makes it a potential candidate for ILE therapy [8].

ILE consists of nanometer‐sized triglyceride droplets emulsified in water by phospholipid surfactant. Initially used to treat local anesthetic agent toxicity, increasing evidence supports its potential role in other lipophilic drug poisonings, including many neuropsychiatric drugs [9]. The most widely accepted mechanism was the “lipid sink” theory whereby lipophilic drugs are sequestered within the intravascular lipid compartment and redistributed to biologically inert tissues. The efficacy of ILE to absorb a toxicant depends on several factors, including the octanol/water partition coefficient (log*P*), the distribution coefficient (log*D*), and drug accommodation capacity [10]. Log*P* accounts for the neutral moiety of the drug, whereas log*D* takes into account both the ionized and nonionized forms of the drug, reflecting more accurately the physiological conditions where both forms of the drug exist in vivo. Interestingly, at the pH of 7.2 observed in our patient, the predicted log*D* of bupropion is approximately 2.2, which is lower than its log*P* (3.47), reflecting partial ionization under physiological conditions, but still consistent with a clinically relevant lipophilic profile. Moreover, partitioning into a triglyceride/phospholipid emulsion may not be fully predicted by the conventional octanol/water distribution coefficient. Finally, the distribution volume of the drug seems also to influence its ability to be sequestered [11].

More recently, the lipid sink concept has now evolved into the “lipid shuttle” theory whereby ILE transiently binds the drug and facilitates its transport from target organs (brain and heart) to tissues involved in storage, metabolism, and elimination of the drug (muscle, adipose tissue, liver, and kidneys). This mechanism may explain the transient increase followed by a decrease in plasma concentrations of lipophilic drugs observed after ILE administration in both animal and human studies [10].

Additional mechanisms have also been hypothesized, including enhanced myocardial fatty acid utilization for ATP synthesis, positive inotropic effects by limiting the toxicant interference with sodium channels and promoting calcium entry through voltage‐dependent calcium channels, activation of protein kinase leading to a cytoprotective signaling cascade, decreased vasodilation through decreased nitric oxide bioavailability, and increased liver shunting [10].

The benefit of ILE in bupropion toxicity remains uncertain. Favorable outcomes reported in some case reports must be interpreted cautiously because of the potential publication bias, as positive outcomes are more likely to be published than negative ones [2]. Although animal studies suggest improved outcomes when ILE is included in resuscitation protocols after bupropion overdose [12], to date no randomized controlled trial in humans has evaluated its efficacy or safety.

In 2016, an expert panel endorsed by several American and European toxicology societies recommended considering ILE as a second‐line treatment for life‐threatening bupropion toxicity after failure of standard treatments [13]. These recommendations were, however, based on a very low quality of evidence, and no recommendation was made regarding dosing or duration of ILE treatment. Potential complications of ILE, including hypertriglyceridemia, pancreatitis, anaphylaxis, and interference with laboratory results, should also be known when assessing the risk/benefit balance of its use. ILE may also interfere with other treatments, such as benzodiazepines, which could explain the recurrence of seizures after ILE administration in our patient.

The multiple serum measurements obtained before, during, and after ILE administration in our patient provide a unique opportunity to explore its potential mechanisms of action. Before ILE, a spontaneous decrease of bupropion levels was observed, whereas metabolite levels continued to rise, reflecting ongoing metabolism. Immediately after the ILE bolus, a 30% increase in bupropion concentration was observed, a phenomenon commonly reported with other lipophilic drug intoxications and consistent with a redistribution from target organs into the intravascular lipid phase. Hydroxybupropion, which is much less lipophilic, remained relatively unaffected but, importantly, did not further increase thereafter. Although this observation could also be explained by redistribution of the parent drug, it raises the hypothesis that ILE administered approximately 8 h after ingestion of bupropion may have limited further metabolism of bupropion.

The dramatic and immediate hemodynamic improvement that occurred immediately after ILE administration is a finding already described in other case reports [14, 15]. The fact that this improvement was observed without any change in the blood levels of hydroxybupropion, which is the main active metabolite of bupropion, suggests mechanisms beyond drug sequestration alone, including direct cardioprotective effects.

Simultaneously, the immediate change in the EEG pattern, characterized by shorter interburst intervals and increased burst amplitude, was consistent with a partial recovery of cortical activity. The temporal relationship with ILE administration raises the hypothesis that ILE may have altered the cerebral distribution of highly lipophilic neuroactive compounds, including diazepam, thereby contributing to the observed partial restoration of cortical electrical activity.

Several limitations must be acknowledged when interpreting our results. First, serum drug concentrations do not reflect tissue levels at target organs. Second, although in nonlipemic blood, the measurement of bupropion and its metabolites reflects the total drug concentration (e.g., both protein‐bound and unbound fractions), in ILE‐treated serum, it remains unknown whether the measured levels accurately represent the total fraction of the drug. Third, no consensus exists regarding optimal ILE dosing or duration. Whether the initial dosing regimen proposed for local anesthetic intoxication can be extended for oral drug poisoning with different pharmacokinetics is unknown. The dosing regimen using a bolus of 1.5 mL/kg, followed by 0.25 mL/kg/min for 3 min, and a continuous infusion thereafter of 0.025 mL/kg/min should reach a plasma level of triglycerides around 1%, which has been suggested to achieve increased inotropic activity and scavenging effects [16]. Given the long elimination half‐life of bupropion and the short half‐life of ILE (13.7 min), a progressive tapering strategy was chosen to prolong therapeutic exposure while avoiding excessive cumulative dose as previously described by others [17]. We believe that, in the absence of robust data supporting one dosing regimen of ILE over another, the pharmacokinetic properties of both the toxicant and of ILE should help guide individualized ILE therapy.

## 4. Conclusions

We presented a case of severe bupropion poisoning with refractory neurological and cardiovascular toxicity. ILE administration was followed by immediate hemodynamic improvement. Serial measurements of bupropion and its metabolites are compatible with intravascular lipid sequestration, whereas the immediate clinical response also suggests additional direct cardioprotective effects of ILE.

## Author Contributions

Francesca Chiara Della Casa: writing—original draft. Vincent Haufroid: laboratory analysis. Kevin Delongie: laboratory analysis. Thomas Coppens: electroencephalogram analysis. Ester Ponzetto: writing. Nicolas De Schryver: supervision and writing—original draft. All authors participated in the writing of the manuscript.

## Funding

No funding was received for this manuscript.

## Disclosure

All authors approved the final version of the manuscript.

## Ethics Statement

The authors confirm that the principal investigator for this paper is Nicolas De Schryver and that he had direct clinical responsibility for patients. Written informed consent was obtained from the patient for the publication of this case report.

## Conflicts of Interest

The authors declare no conflicts of interest.

## Data Availability

The data that support the findings of this study are available from the corresponding author upon reasonable request.
